# Simulation-based validation of spatial capture-recapture models: A case study using mountain lions

**DOI:** 10.1371/journal.pone.0215458

**Published:** 2019-04-19

**Authors:** J. Terrill Paterson, Kelly Proffitt, Ben Jimenez, Jay Rotella, Robert Garrott

**Affiliations:** 1 Department of Ecology, Montana State University, Bozeman, Montana, United States of America; 2 Montana Department of Fish, Wildlife and Parks, Bozeman, Montana, United States of America; United States Department of Agriculture, UNITED STATES

## Abstract

Spatial capture-recapture (SCR) models have improved the ability to estimate densities of rare and elusive animals. However, SCR models have seldom been validated even as model formulations diversify and expand to incorporate new sampling methods and/or additional sources of information on model parameters. Information on the relationship between encounter probabilities, sources of additional information, and the reliability of density estimates, is rare but crucial to assessing reliability of SCR-based estimates. We used a simulation-based approach that incorporated prior empirical work to assess the accuracy and precision of density estimates from SCR models using spatially unstructured sampling. To assess the consequences of sparse data and potential sources of bias, we simulated data under six scenarios corresponding to three different levels of search effort and two levels of correlation between search effort and animal density. We then estimated density for each scenario using four models that included increasing amounts of information from harvested individuals and telemetry to evaluate the impact of additional sources of information. Model results were sensitive to the quantity of available information: density estimates based on low search effort were biased high and imprecise, whereas estimates based on high search effort were unbiased and precise. A correlation between search effort and animal density resulted in a positive bias in density estimates, though the bias decreased with increasingly informative datasets. Adding information from harvested individuals and telemetered individuals improved density estimates based on low and moderate effort but had negligible impact for datasets resulting from high effort. We demonstrated that density estimates from SCR models using spatially unstructured sampling are reliable when sufficient information is provided. Accurate density estimates can result if empirical-based simulations such as those presented here are used to develop study designs with appropriate amounts of effort and information sources.

## Introduction

Estimates of population size are of fundamental importance for understanding and managing populations of wild animals. In response to the imperfect detection of individual animals in field studies, ecologists have developed a diverse set of tools to account for heterogeneity in detection [1–4]. However, certain species such as large carnivores present persistent methodological and analytical obstacles to abundance estimation given their elusive nature, low densities, large ranges and potential for territoriality [5–7]. These ecological characteristics frequently result in low probabilities of capture and induce spatial heterogeneity in capture probabilities among individuals, both of which challenge traditional capture-recapture models [8–11]. Moreover, the large-scale movements of individuals relative to a fixed grid of trapping locations complicates the estimation of sampling area, which is required to convert an abundance estimate into a density estimate, a more useful metric for comparing populations across different geographic extents [8,12]. Yet, despite these challenges in estimating density, accurate estimates are required for wildlife management and conservation for the purposes of setting harvest quotas, the integrated management of predator and prey species, the conservation of threatened and endangered species, and to mitigate human-wildlife conflict.

Spatial capture-recapture models (SCR) are a relatively new method of estimating density that attempt to resolve the shortcomings of traditional capture-recapture approaches by explicitly modeling the spatial organization of individuals relative to spatially referenced trapping locations [8,13]. In this approach, detection probability is modeled as a function of the distance between the locations of traps and activity centers for various individuals, as well as trap- and individual-specific covariates. In doing so, SCR models account for the different exposure of individuals to detection due to their spatial distribution on the landscape. By explicitly incorporating the spatial arrangement of individuals, SCR models resolve a major drawback of non-spatial capture recapture models: the ad-hoc estimation of density using the addition of a buffer around the trapping grid to account for differential exposure of individuals [3,12,14].

However, despite the advances in density estimation associated with SCR models, the approach requires careful application. Theoretical and empirical work suggests that SCR models require large numbers of individuals and spatially distributed recaptures to accurately and precisely estimate model parameters and, therefore, abundance and density [15,16]. This problem is exacerbated as the number of sources of heterogeneity in the exposure of individuals to capture increases, e.g., among-individual differences in the probability of capture due to sex, age class, or social status [10,17–19]. Estimating the parameters of more complex models that account for additional sources of heterogeneity requires still more information, which can be problematic given the practicalities of sampling. Because species to which SCR models are frequently applied are often elusive and occur at low density, probabilities of detection can be low such that resulting datasets are sparse and contain insufficient information to accurately estimate the parameters of SCR models [15,18,20].

A variety of non-invasive sampling methods with different detection efficiencies have been used in a SCR framework, including camera traps [13,21,22], hair snares [23,24], and scat detection [25–27]. For species in which visual identification is difficult, DNA-based identification methods such as hair snares, biopsy darting, or scat sampling are required. One technique that has been shown to greatly increase the probability of detection of animals is the use of scent dogs, wherein teams of specialized dogs are used in an organized search for evidence of animals [25]. However, direct search effort using scent-detection dogs is inherently unstructured. In contrast to the identification of individuals at fixed trapping locations or along fixed transects, encounters result from scent-detection dogs following airborne scent or ground-based scent, making precise delineation of where sampling occurs difficult. To resolve the locations of detections and non-detections in this type of unstructured sampling approach, previous work has conceptualized a coarse, post-hoc grid of trapping cells overlying the study area [25]. The center of each grid cell serves as the hypothetical trapping location such that the locations of detections and non-detections, as well as the associated search effort, can be spatially referenced.

A parallel development in SCR methods in recent years has been the inclusion of additional sources of information [28–32]. Where data sets are sparse and practical realities prohibit increasing the probability of detection, alternative sources of information can improve the accuracy and precision of parameter estimates [29,32]. For example, previous results from a SCR study on mountain lions (*Puma concolor*) in Montana demonstrated that including the locations of harvested individuals as if they arose from the spatial trapping effort greatly increased sample size, resulting in a dramatic decrease in estimated densities and reduction in uncertainty (improvement in precision) [33]. More formally, the hierarchical formulation of SCR models facilitates the integration of data from different encounter types by allowing model parameters to be shared among processes [34]. For example, telemetry data from collared individuals provides additional information on animal movement and can improve estimation of model parameters using an integrated likelihood provided space use is similar between individuals observed by the encounter process and collar data [32,34,35].

Despite the development of new sampling methods and data-integration techniques, little guidance exists on how to practically implement a SCR study using these new methods such that the resulting model estimates of abundance/density are reliable. Recent work has suggested that these models should perform well given sufficient information on space-use patterns and detection [20], but that insufficient information arising from among-individual heterogeneity and generally low probabilities of detection, or the specifics of sampling methods, can result in strongly biased and imprecise density estimates [18,19,22,36]. Therefore, it is critical to understand potential sources of bias so as to be able to make recommendations for study design. Yet, we know of only three cases where SCR models have been tested against populations of a known size, with apparently contrasting conclusions about sources and direction of bias [19,22,36]. Most work has focused on simulation-based validation, which has been criticized due to the inability to fully consider the breadth of processes that lead to data [19]. Moreover, bias in SCR models arises from processes that are likely to be species- and even study-specific, such that increasing biological realism in simulation-based validation reduces generality.

Here, we present an approach for simulation-based validation that uses empirical studies to understand the consequences of sparse data arising from low detection probabilities and the implications of including additional information on density estimates. We were motivated by the recent use of SCR models to estimate the density of mountain lions in western Montana using a spatially unstructured sampling design relying primarily on the use of scent-detection dogs [33]. That work was notable in that it demonstrated the inhomogeneous distribution of individual activity centers across the landscape, and suggested mountain lion density was strongly associated with underlying habitat characteristics. Moreover, the work highlighted how the inclusion of samples from harvested individuals improved inference by both reducing density estimates and improving precision. However, SCR model performance under these conditions is essentially unknown. We therefore conducted simulations to assess model performance in the presence of a heterogeneous distribution of animals across the landscape and differences in space use between sexes. Our simulations focused on evaluating the consequences of including additional information from a simulated harvest process and/or telemetry information from collared individuals. Finally, we demonstrate how our approach can yield practical advice to guide the allocation of sampling effort and intensity and to help inform study designs for other species-specific SCR models.

## Materials and methods

### Simulation design

Our work assessed model performance for density estimates of mountain lions in western Montana and focused on the consequences of varying search effort and the inclusion of additional sources of information from harvest and telemetry. The design of the simulations and the choices of the underlying parameters that govern the data-generating process were based on previous empirical work on this species in this area [33]. The major components of the simulation process were: (1) the definition of the landscape including underlying structure, (2) the relationship between the structure in the landscape and the distribution of individual activity centers (the spatial point process), and (3) the relationship between the distribution of individual activity centers and the probability of encounter. This diversity of components in hierarchical SCR models renders an exhaustive treatment of possible simulation scenarios practically challenging given the computational requirements, and our work was restrained to assessing the consequences of varying search effort and additional information in the encounter process (the third component, above).

#### Defining the landscape

**Fig 1:**
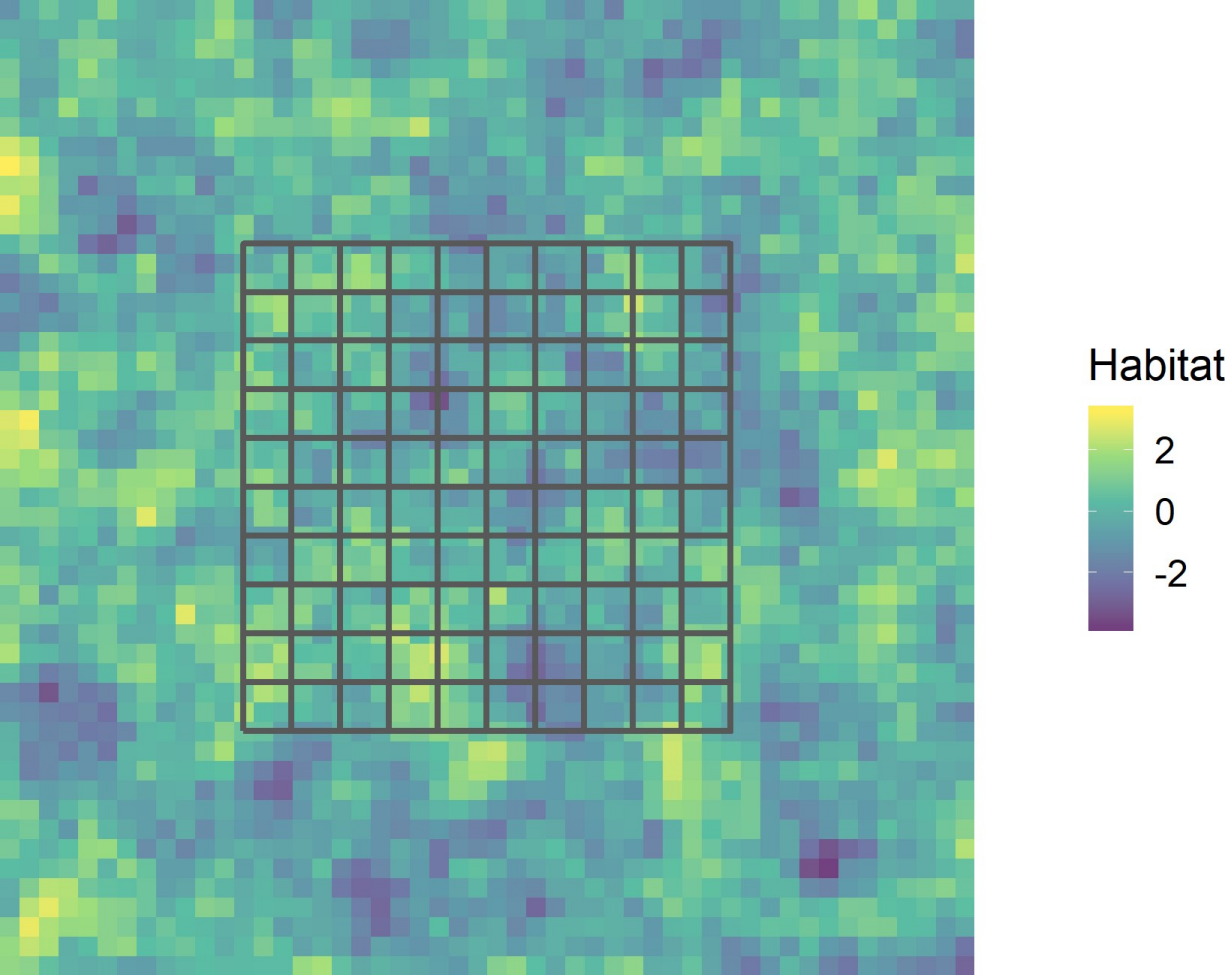
Simulated 10,000 km^2^ landscape. Our simulated landscape was comprised of 2,500 2km x 2km landscape cells and defined by a spatially correlated habitat covariate ranging from poor habitat (negative) to good habitat (positive). The study area was defined by a trapping grid comprised of 100 5km x 5km grid cells centered on the landscape (black).

We based our simulations on a 2,500 km^2^ (50km x 50km) study area, defined by a grid of 100 non-overlapping 5km x 5km trapping cells. This trapping grid was centered in a 10,000 km^2^ simulated landscape (100km x 100km) defined by a grid of 2,500 non-overlapping 2km x 2km cells, each of which was potentially the center of a home range, or activity center, for an individual. The sizes of the study area and trapping grid, as well as the sizes of the underlying trapping grid cells and idealized home range centers, were based on the prior empirical work [33]. Prior work also demonstrated a strong relationship between the spatial arrangement of individuals and habitat structure as indexed by the values of a separately estimated resource selection function [33,37]. Therefore, we generated a spatially autocorrelated habitat covariate for each landscape grid cell (Habitat_*x*,*y*_ where *x*,*y* represents the center of each cell) on the landscape using a kriging model of correlated random noise [29] (Fig 1).

#### Defining the spatial point process

We then generated spatial structure in our population by simulating the locations of activity centers using an inhomogeneous point process. Specifically, we probabilistically assigned an activity center to individuals such that the expected number of activity centers in any given area was a function of the underlying habitat covariate. We defined the inhomogeneous point process by defining the intensity in each landscape cell as *μ*_*x*,*y*_ = exp(−4 + 0.5Habitat_*x*,*y*_)(4 km^2^), scaling the relationship by the area of each cell, 4 km^2^. Finally, to simulate the locations of activity centers such that they were correlated to the underlying habitat covariate, we used rejection sampling to probabilistically assign an activity center to each individual, *s*_*i*_, based on the relative intensity value in each grid cell [29,38]. These activity centers were assigned independently, i.e., more than one mountain lion was allowed to be assigned to each activity center. Prior work has demonstrated that home ranges of mountain lions demonstrate considerable overlap between and among sexes, suggesting this simplification is well-justified [39,40]. We first simulated the distribution of activity centers using a fixed population sizes of *N* = 200 individuals (100 males and 100 females corresponding to a density over the landscape of 2 individuals per 100 km^2^). This is at the low end of densities in this region [33,41], and represents a worst-case scenario of maximizing search effort and additional information to generate reliable density estimates. To evaluate model performance under higher densities, we also generated distributions of activity centers for a fixed population of *N* = 400 individuals (200 males and 200 females).

#### Defining the encounter processes

Previous empirical work utilized observations of individuals that resulted from both organized search effort and harvest [33], and current work in the same system is evaluating the impact of including information from collared individuals on density estimates. Therefore, we simulated three types of data, using separate processes for: (1) observations from spatially unstructured sampling (the encounter process), (2) from the harvest of individuals, and (3) to generate telemetry data. For the encounter process, we simulated encounters of each individual *i* (*i* = 1, …, 200) in each grid cell *j* (*j* = 1, …, 100) during each occasion *t* (*t* = 1, 2, 3, 4), corresponding to the four month period from December to March previously used in this study area [33], using a Binomial model:

$${encounter}_{i,j,t}∼Binomial(p_{i,j,t},1)$$

We used the exponential model to define how the probability of encounter decays with distance from an activity center such that the probability of encounter of individual *i* in trapping cell *j* during occasion *t* is given as:

$$cloglog(p_{i,j,t})=\beta_{0}^{encounter}+\beta_{eff}^{encounter}*\log\left({effort}_{j,t}\right)-\frac{1}{2\sigma_{sex}^{2}}*d(x_{j},s_{i})$$

wherein effort_*j*,*t*_ is the effort expended in cell *j* during occasion *t*, *σ*_*sex*_ is a sex-specific scale parameter that regulates how the probability of encounter declines with distance, d(*x*_*j*_,*s*_*i*_) is the Euclidean distance between the center of the trapping cell, *x*_*j*_, and the activity center of individual, *s*_*i*_, and cloglog is the complementary log-log link.

We also simulated a harvest process for individuals in the system using a similar structure to the encounter process. The harvest of an individual *i* in grid cell *j* during occasion *t* was modeled as a Binomial process,

$${harvest}_{i,j,t}∼Binomial(h_{i,j,t},1)$$

wherein the probability of harvest shares the structural parameters *σ*_*sex*_ with the encounter process and is given as:

$$cloglog(h_{i,j,t})=\beta_{0}^{harvest}-\frac{1}{2\sigma_{sex}^{2}}*d(x_{j},s_{i})$$

and used $\beta_{0}^{harvest}=-2.25$, which corresponds to a baseline probability of harvest (when d(*x*_*j*_,*s*_*i*_) = 0) in each trapping grid cell of ~ 0.10. This roughly approximates actual numbers of harvested mountain lions previously reported [33], though the assumption of a constant baseline probability of harvest (i.e., lacking spatial structure) is a simplification.

Finally, we simulated daily telemetry data. For every individual *i*, we simulated the use frequencies for each cell in the landscape, *m*_*i*,*k*_, *k* = 1,…,2500, as a multinomial process [29]:

$$m_{i}∼Multinomial(R,\pi_{i})$$

with R = 30 (simulating daily observations for one month) and where the probability vector *π*_*i*_ is calculated as

$$\pi_{i,k}=\frac{exp(\frac{-1}{2\sigma_{sex}^{2}}*d(x_{k},s_{i}))}{\sum_{k=1}^{2500}exp(\frac{-1}{2\sigma_{sex}^{2}}*d(x_{k},s_{i}))}$$

such that the probability of use declines with distance from the activity center of an individual using the same function of distance used in the encounter and harvest processes. It is important to point out that all three processes shared parameters. This allowed the use of these additional data to aid estimation, but also made the tacit assumption that these parameters are consistent across the different processes [35].

The parameters for the data-generating processes ($\beta_{0}^{encounter},\beta_{0}^{harvest},\beta_{eff},\sigma_{male},\sigma_{female}$) are species-, study-area-, and method- specific. Using results from previous work in this system [33,41], we generated encounter histories using $\beta_{0}^{encounter}=-5$, *β*_*eff*_ = 1, log(*σ*_*male*_) = −0.65 and log(*σ*_*female*_) = −0.85. For the space-use parameters, these correspond to distances that are in units of 5 km, of the distance between the centers of adjacent trapping cells. Such scaling is useful to aid convergence for both Bayesian- and maximum-likelihood-based methods.

### Simulation scenarios

Our primary goal was to assess SCR model performance (the combination of accuracy and precision) for spatially unstructured sampling designs at the low end of reported densities (*N* = 200, or 2 individuals per 100 km^2^) in the presence of variable effort. Spatially unstructured sampling designs are also potentially subject to bias in estimates if the search effort is correlated to animal density, i.e., effort is biased towards areas of higher animal density [25,41]. Therefore, we evaluated model performance in the presence of variable effort, sparse data, and this induced correlation. Our second goal was to assess the benefits of integrating additional sources of information from harvest and telemetry on resulting density estimates.

**Fig 2:**
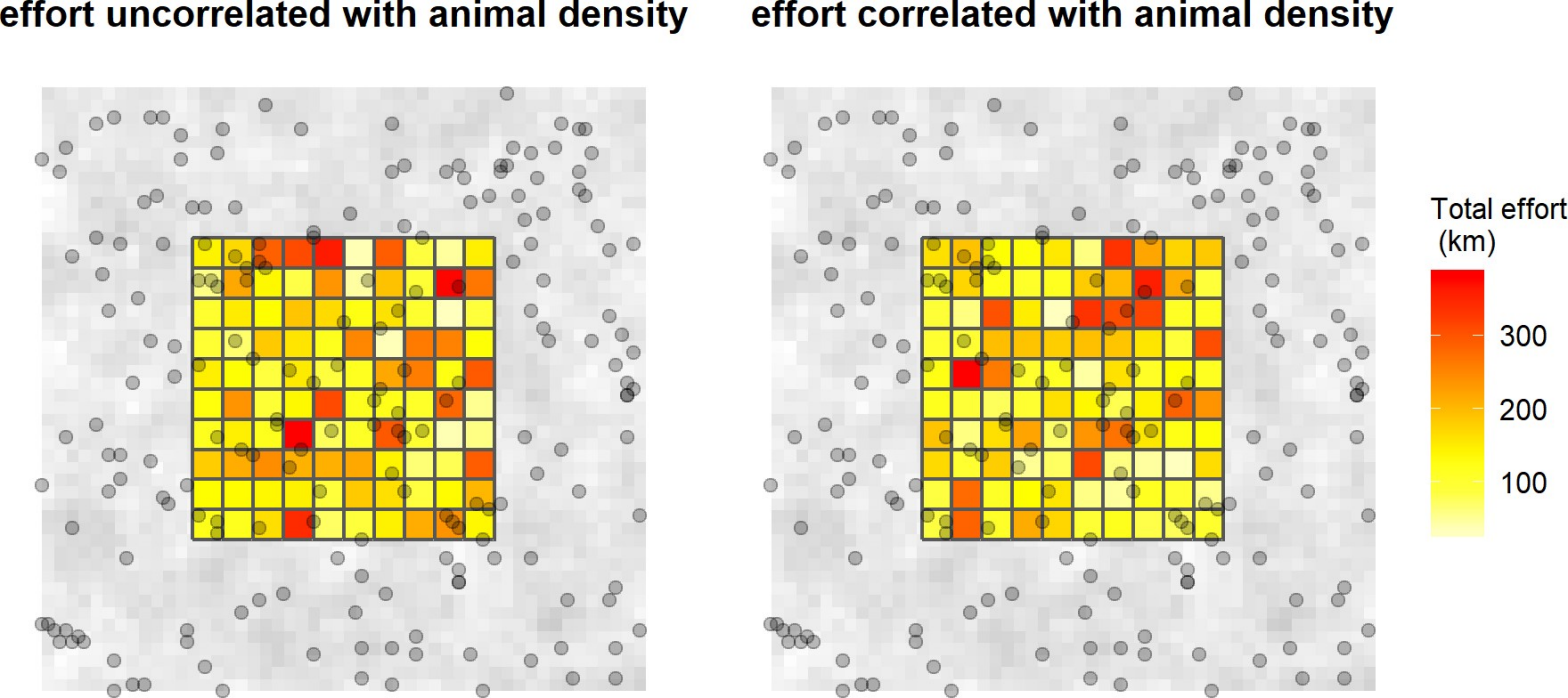
Simulated effort. To illustrate the distribution of simulated effort across the trapping grid cells, we took a single realization from a simulation with moderate effort (4,000 km of effort per sampling occasion = 16,000 km total, no correlation with animal density, left panel) and compared it to a single realization with the same effort, but with a correlation between density and effort (right panel). The spatially correlated landscape and locations of activity centers (dots) are shown in the background.

For spatially unstructured sampling, encounters are the result of direct search effort (kilometers of search effort expended in each grid cell during each occasion). To assess the amount of bias induced by sparse data we simulated encounters under three levels of effort corresponding to low, medium, and high baseline probabilities of encounter, i.e., the probability of encounter when an animal’s activity center is coincident with the center of a trapping grid. These levels were defined using 2,000 km, 4,000 km, and 8,000 km of effort per occasion. First, we generated a random level of effort for each cell of a trapping grid in each occasion by taking the total effort (2,000, 4,000, or 8,000 km per occasion) and multiplying it by a random draw from a Dirichlet distribution (all parameters equal to 1, or a flat Dirichlet distribution), which is a straightforward way to randomly assign a total to a series of trapping cells. Second, to assess the bias induced by a correlation between effort and the density of activity centers, we repeated the above procedure but used the number of activity centers in each trapping grid to weight the parameters of the Dirichlet distribution, i.e., a trapping grid cell with no activity centers had a value of 1, a trapping grid cell with 1 activity center had a value of 2, etc. This induced a strong correlation between animal density and effort (e.g., Fig 2). All combinations of effort (three levels) and correlation (two levels: correlated and uncorrelated effort) resulted in six scenarios.

Next, to evaluate the consequences of including extra information for each scenario, we estimated the density of individuals using four models with increasing levels of information: 1) a model using encounters from the spatially unstructured sampling alone, 2) a model combining encounter and harvest processes, 3) a model combining encounter, harvest and telemetry information from 2 males and 2 females (4 total), and 4) a model combining encounter, harvest, and telemetry information from 4 males and 4 females (8 total). In each case, we estimated the parameters of the data-generating model, i.e. our goal was not to assess the consequences of model mis-specification, but to evaluate model performance as the amount of information in the data set increased. Importantly, the locations of harvested individuals were treated as if they resulted from the search process, and the effort covariate for the trapping cell in which the individual was harvested was “borrowed” from the spatial capture-recapture search effort, similar to previous work in this system [33]. When an animal was harvested, subsequent trapping occasions were removed from the likelihood (e.g., an animal harvested during the second trapping occasion was unavailable for detection during occasions 3 and 4). When telemetry information was incorporated, it was assumed to be independent from the spatial capture-recapture data. This assumption simplifies computation and introduces negligible bias to resulting density estimates [29].

### Model estimation and performance

We used a maximum-likelihood based method derived from the oSCR package [42] implemented in the R environment [43] and based on the underlying spatial capture-recapture model that allows the integration of telemetry information [34] to estimate the parameters of the data-generating model. For each simulation, we generated data under each of the six scenarios and then used those data to estimate density from each of our four models, which resulted in 24 model runs per simulation. Computation time was prohibitively slow with a direct application of the oSCR package. To increase the speed of model estimation we used custom scripts that streamlined the model formulation and estimation and used the NIMBLE programming language for additional efficiency gains (S1 File) [44]. We assessed model performance using estimated means, standard deviations, 2.5% and 97.5% percentiles (95% density interval), the coefficient of variation (CV) and relative bias (RB).

## Results

Our primary goal was evaluating model performance in the worst-case scenario of low animal density (*N* = 200 individuals, 2 per 100km^2^) and we focused on these results below. However, we also compared and contrasted these results to model performance at a higher density of *N* = 400 individuals (4 per 100 km^2^).

### Simulated spatial capture-recapture data

**Fig 3:**
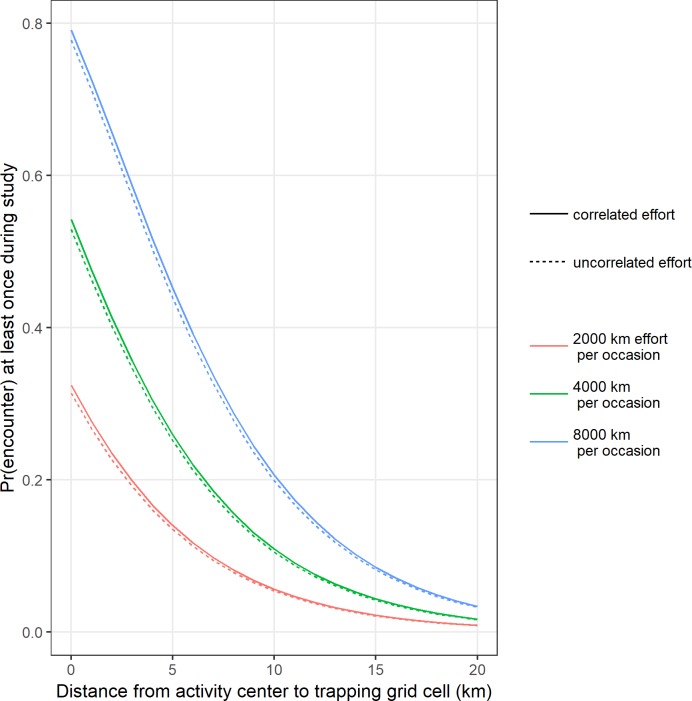
Search effort and the probability of encounter. The relationship between the total effort per occasion (2,000 km, 4, 000 km, or 8,000 km), and the resulting median probability of encountering an individual at least once in a trapping grid cell as a function of distance from the activity center during a 4-occasion study. Show for males only for clarity.

The combination of varying effort (2000 km, 4000 km, 8000 km of total effort per occasion) and the correlation between animal density and effort resulted in six data-generating scenarios. We generated 200 data sets under each scenario and summarized them by how the total effort translated into a median effort per cell and per occasion, cell-specific baseline encounter probabilities and the induced correlation with animal density (Table 1). The per-cell, per-occasion effort is directly related to the baseline probability of encounter in each trapping grid cell, which then declined as a function of distance from activity centers according to our model for the probability of encounter (Fig 3).

**Table 1:** Summary statistics for simulated data. Datasets were generated under three levels of search effort per sampling occasion (2,000 km, 4,000 km, and 8,000 km) that was uncorrelated or correlated with animal density, resulting in six data-generating scenarios. The baseline probability of detection in each scenario (median and (2.5%, 97.5%) percentiles) corresponded to the probability of observing an individual whose activity center is coincident with the center of the trapping grid cell.

	2000 km effort per occasion		4000 km effort per occasion		8000 km effort per occasion	
	uncorrelated	correlated	uncorrelated	correlated	uncorrelated	correlated
	Scenario 1	Scenario 2	Scenario 3	Scenario 4	Scenario 5	Scenario 6
effort per cell per occasion (km)	13.95 (12.70, 15.31)	14.68 (12.79, 16.18)	28.11 (25.10, 31.10)	28.95 (26.26, 31.60)	56.08 (50.42, 61.58)	58.08 (50.42, 61.58)
baseline Pr(encounter)	0.09 (0.08, 0.10)	0.09 (0.08, 0.10)	0.17 (0.16, 0.19)	0.18 (0.16, 0.19)	0.31 (0.29, 0.34)	0.32 (0.29, 0.35)
correlation with animal density	0.01 (-0.18, 0.18)	0.76 (0.66, 0.83)	-0.01 (-0.18, 0.18)	0.76 (0.67, 0.84)	0.00 (-0.17, 0.18)	0.76, 0.65, 0.82)

Each simulated data set was comprised of data from the encounter process alone, data that combined encounter and harvest, and data that combined observation, harvest and two levels of telemetry information. Including information from a harvest process typically increased the number of individuals in the data set, and for each scenario we summarized the data sets for the encounter process alone, and the combination of encounter and harvest, using the number of unique individuals (n), the number of recaptures (recap), and the number of spatial recaptures (spatial.recap), or the number of different cells in which individuals were captured (Table 2).

**Table 2:** Summary statistics (median and (2.5%, 97.5%) percentiles) for the generated data sets (N = 200). For each scenario we generated 200 data sets using the encounter process alone, and the encounter process combined with a harvest process. We summarized them by the number of unique individuals seen in the data (n), the number of recaptures per individual (recap), and the number of cells in which an individual is captured (spatial.recap).

		2000 km effort per occasion		4000 km effort per occasion		8000 km effort per occasion	
		uncorrelated	correlated	uncorrelated	correlated	uncorrelated	correlated
		Scenario 1	Scenario 2	Scenario 3	Scenario 4	Scenario 5	Scenario 6
Encounter alone	n	22 (14, 33)	26 (16, 36)	37 (25, 47)	40 (28, 52)	51 (35, 65)	52 (40, 67)
	recap	1.28 (1.06, 1.54)	1.35 (1.14, 1.63)	1.57 (1.34, 1.90)	1.72 (1.46, 2.08)	2.10 (1.80, 2.52)	2.40 (1.98, 2.85)
	spatial.recap	1.23 (1.05, 1.46)	1.26 (1.08, 1.48)	1.46 (1.22, 1.72)	1.49 (1.27, 1.76)	1.85 (1.59, 2.16)	1.93 (1.67, 2.25)
Encounter and Harvest	n	31 (18, 47)	35 (19, 50)	44 (29, 59)	45 (33, 61)	55 (37, 71)	57 (41, 70)
	recap	1.32 (1.10, 1.56)	1.39 (1.17, 1.65)	1.61 (1.36, 1.87)	1.77 (1.49, 2.09)	2.17 (1.87, 2.55)	2.47 (2.04, 2.91)
	spatial.recap	1.26 (1.07, 1.45)	1.29 (1.11, 1.49)	1.48 (1.26, 1.70)	1.54 (1.30, 1.77)	1.89 (1.65, 2.21)	1.97 (1.73, 2.29)

### Density estimates using the encounter process alone

**Fig 4:**
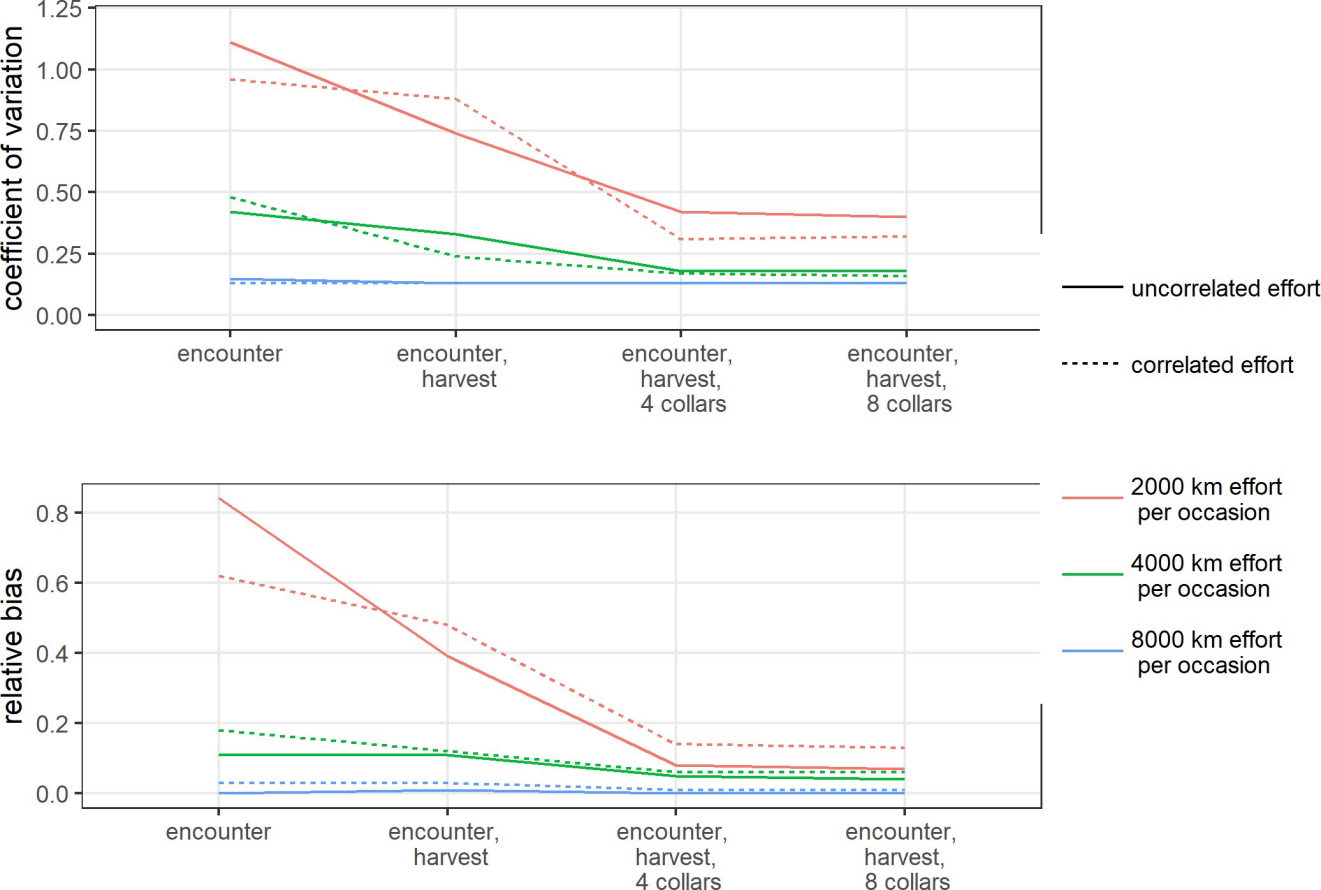
Model performance. Model performance was assessed by the coefficient of variation (CV) and relative bias (RB) for different combinations of effort (2,000 km, 4,000 km and 8,000 km of effort per occasion), correlation with animal density, and sources of additional information (harvest and collars).

**Fig 5:**
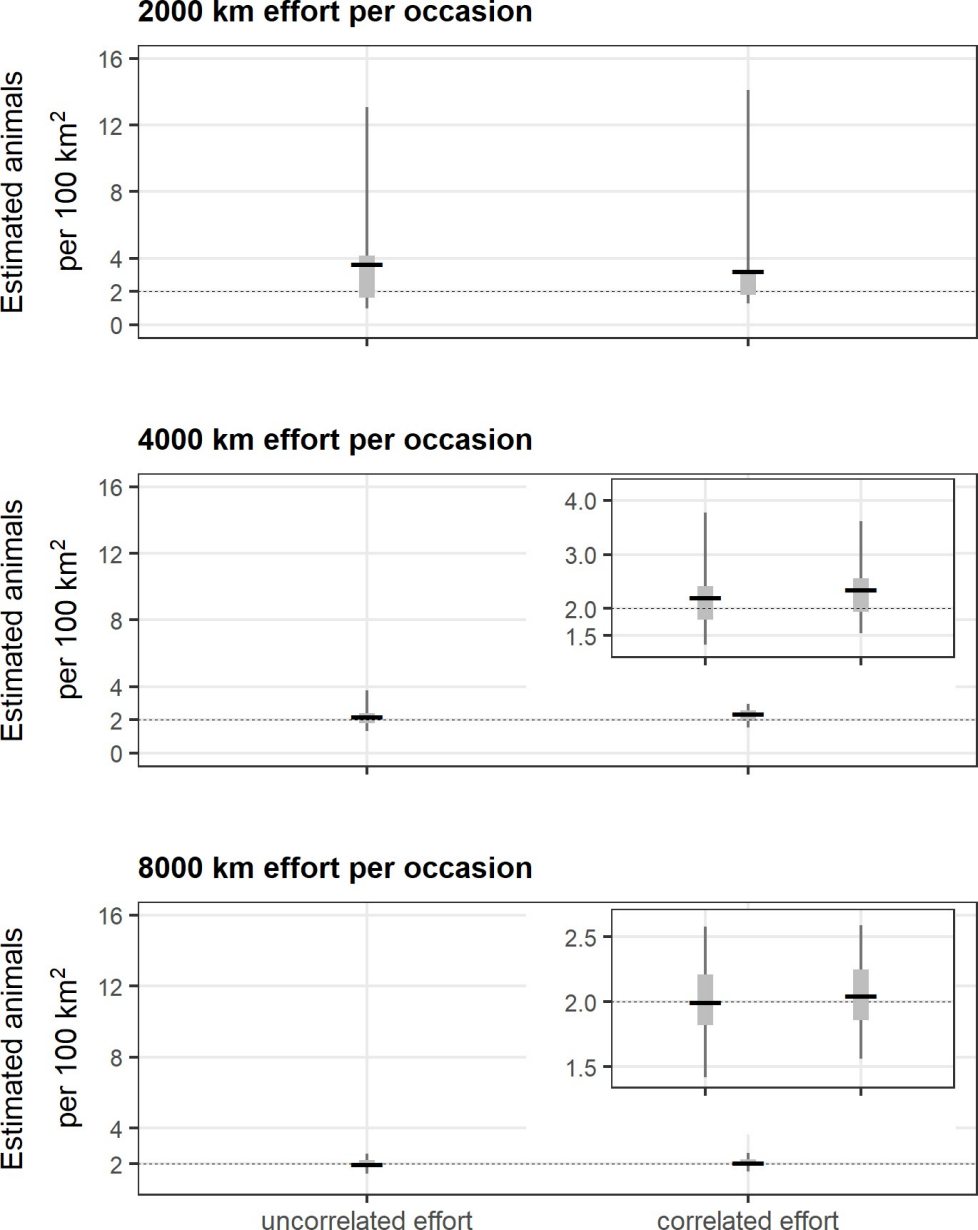
Density estimates and search effort. Estimates of animal density resulting from the encounter process alone for each level of search effort (thin line = 2.5% to 97.5% percentiles; gray rectangle = 25% to 75% percentiles; horizontal line = mean). The true density was 2 individuals per 100 km^2^. The inset graphs show the same data with a y-axis that is rescaled to better represent estimates.

The amount of effort during each sampling occasion had a dramatic impact on how well resulting density estimates compared to the true abundance of 2 individuals per 100 km^2^ (Figs 4 and 5). For the encounter process alone with effort uncorrelated to animal density (scenario 1, median correlation = 0.01, 95% = [-0.18, 0.18]) (Table 1), a low amount of effort (2000 km per sampling occasion) translated into a low baseline probability of encounter (median = 0.09, 95% = [0.08, 0.10]), low number of observed individuals (median = 22, 95% = [14, 33]), and low numbers of recaptures (median = 1.28, 95% = [1.06, 1.54]) and spatial recaptures of individuals (median = 1.23, 95% = [1.05, 1.46]) (Table 2). These sparse data sets yielded biased and imprecise estimates which translated into poor estimation of animal density (coefficient of variation (CV) = 1.11, relative bias (RB) = 0.84) (Figs 4 and 5, S1 Table). As the total amount of effort increased to 4000 km (scenario 3), increased encounter probabilities (median = 0.17, 95% = [0.16, 0.19]) resulted in more observed individuals (median = 37, 95% = [25, 47]), higher numbers of recaptures (median = 1.57, 95% = [1.34, 1.90]), and more spatial recaptures (median = 1.46, 95% = [1.22, 1.72]). The increased amount of information due to increased effort resulted in less biased and more precise estimates and greatly improved biological inference (CV = 0.42, RB = 0.11) (Figs 4 and 5, S1 Table). As effort further increased (scenario 5), model performance continued to improve such that with 8000 km of effort, density estimates were essentially unbiased and precise (CV = 0.15, RB = 0.00) (Figs 4 and 5, S1 Table).

As predicted, when search effort was correlated with the underlying density of activity centers the resulting density estimates were biased high (Figs 4 and 5, S1 Table). However, we found that the magnitude of this bias decreased with increasing effort and was negligible at the highest level of effort. Compared to what was obtained from the same level of effort when it was uncorrelated to density, 2,000 km of search effort that was correlated with animal density (scenario 2, median correlation = 0.76, 95% = [0.66, 0.83]), yielded simulated data sets that had slightly more capture-recapture information (median observed individuals = 26, 95% = [16, 36]: median recaptures = 1.35, 95% = [1.14, 1.63]: median spatial recaptures = 1.26, 95% = [1.08, 1.48]), but the resulting estimates were similarly biased and imprecise (CV = 0.96, RB = 0.62) (Figs 4 and 5, S1 Table). This pattern was consistent as the amount of effort increased, though the relative difference in bias between uncorrelated and correlated search-effort scenarios diminished as higher probabilities of encounter yielded more informative data sets. Density estimates resulting from 8,000 km of correlated search effort had a bias and precision (scenario 6: CV = 0.13, RB = 0.03) roughly equal to that resulting from uncorrelated search effort (scenario 5: CV = 0.15, RB = 0.00) (Figs 4 and 5, S1 Table).

We observed the same pattern of improvement in model performance with increased effort when the number of individuals was *N* = 400 (4 individuals per 100 km^2^) (S2 Table, S1 Fig). However, the higher density resulted in more informative data sets such that model performance at lower effort (e.g., 4000 km of uncorrelated effort: CV = 0.18, RB = -0.01) was comparable to model performance under high effort when the density was lower (*N* = 200, 8000 km of uncorrelated effort: CV = 0.15, RB = 0.00) (S2 Table, S1 Table). Moreover, the bias that was introduced when search effort was correlated to animal density persisted under the higher density for 2,000 km (uncorrelated effort: RB = 0.24, correlated effort: RB = 0.31) and 4,000 km of search effort (uncorrelated effort: RB = -0.01, correlated effort: RB = 0.07). This bias was negligible at higher effort (8,000 km; uncorrelated effort: RB = -0.02, correlated effort: RB = 0.03).

### Consequences of increasing additional information

**Fig 6:**
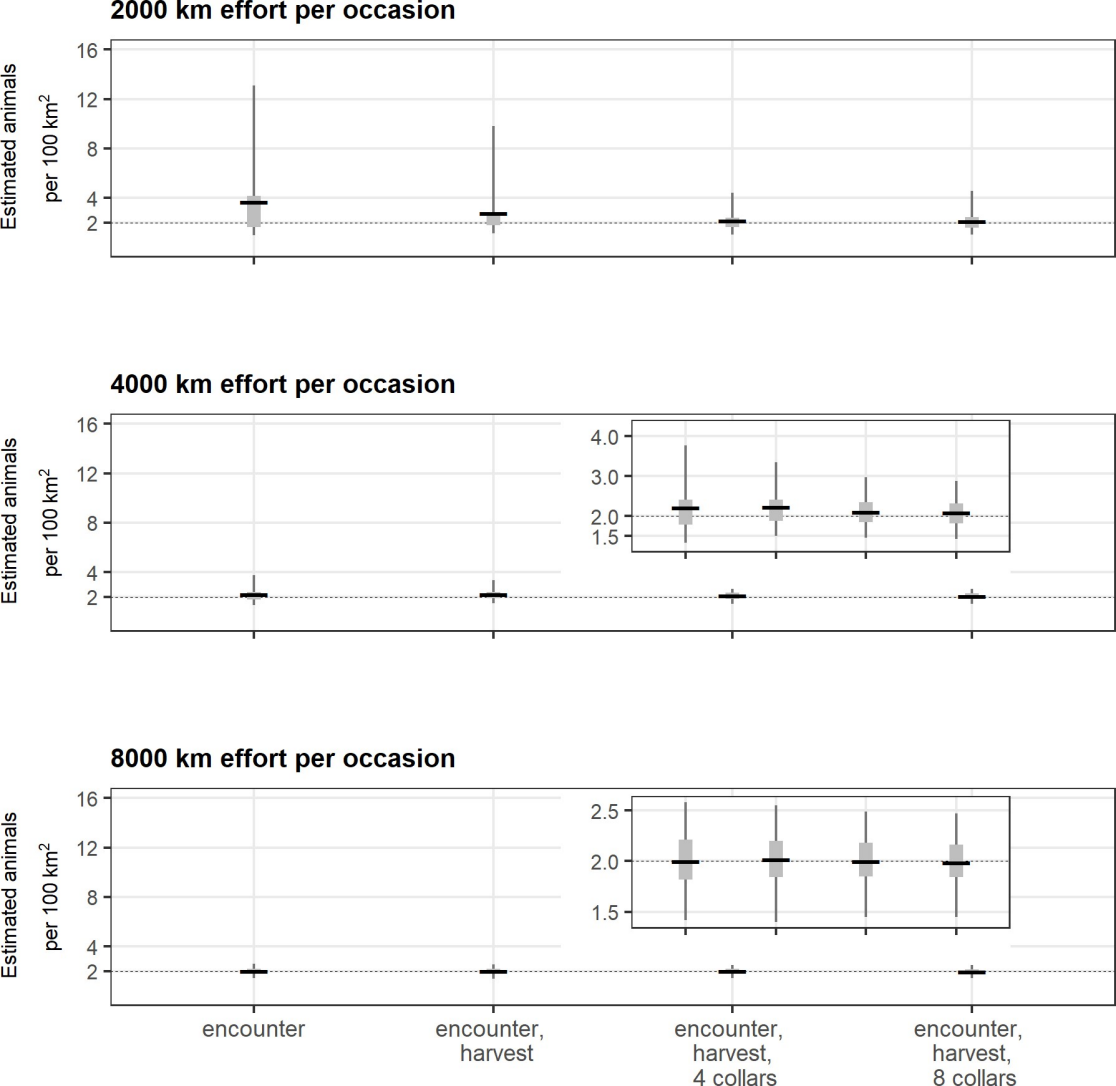
Density estimates and additional information. Consequences of including additional information on estimates of animal density for each level of search effort that was uncorrelated with animal density (thin line = 2.5% to 97.5% percentiles; gray rectangle = 25% to 75% percentiles; horizontal line = mean). The true density was 2 individuals per 100 km^2^. The inset graphs show the same data with a y-axis that is rescaled to better represent estimates.

For 2,000 km of uncorrelated search effort (scenario 1), including data from harvested individuals yielded better model performance with decreased bias and improved precision of density estimates (CV = 0.74, RB = 0.39) relative to estimates obtained from the encounter process alone (CV = 1.11, RB = 0.84) (Figs 4 and 6, S1 Table). Model performance continued to improve when telemetry data from 4 collars were included (CV = 0.42, RB = 0.08). Adding telemetry data from 8 rather than 4 collars resulted in only a marginal additional improvement in density estimates, and estimates were still biased high (Figs 4 and 6, S1 Table). Moreover, the addition of information from harvest and telemetry did not resolve the bias introduced with correlated search effort (Fig 4, S1 Table).

For 4,000 km of uncorrelated search effort (scenario 3), including information from harvested individuals had comparatively minor impact on both bias and precision relative to results from the encounter process alone (Figs 4 and 6, S1 Table). However, including telemetry information from 4 collars improved model performance and resulted in density estimates that were less biased and more precise (CV = 0.18, RB = 0.05) (Figs 4 and 6, S1 Table). There was no further improvement when telemetry information from 8 rather than 4 collars was provided. In contrast to the low-effort scenarios, including additional information from harvest or telemetry partially ameliorated the bias introduced with correlated moderate search effort (scenario 4) even as bias and precision improved overall (Figs 4 and 6, S1 Table).

Including additional information at the highest level of effort did not appreciably improve density estimates. For 8,000 km of uncorrelated search effort (scenario 5), data generated from the encounter process alone resulted in essentially unbiased and precise estimates (Figs 4 and 6, S1 Table). Even with information from harvested individuals and telemetry information from 8 collars, density estimates (CV = 0.13, RB = 0.00) were comparable to those from the encounter process alone (CV = 0.15, RB = 0.00). The positive bias introduced by correlated search effort was negligible at this highest level of effort across the different combinations of information (Fig 4, S1 Table).

Including additional information from harvest and from telemetry also improved model performance at higher densities (*N* = 400, 4 individuals per 100km^2^), although primarily for only the low search effort (2,000 km) scenarios (S2 Table, S2 Fig). At 2,000 km of search effort, including information from harvested individuals resulted in marginal improvement over estimates from the encounter process alone (encounter alone: CV = 0.69, RB = 0.24; encounter and harvest: CV = 0.54, RB = 0.21). However, adding information from 4 collars resulted in improved model performance (encounter, harvest and 4 collars: CV = 0.28, RB = 0.03), although adding information from 8 collars resulted in no additional improvement in performance. In contrast to model performance at lower densities, adding additional information from harvest or collars did not appreciably improve estimates at either 4,000 km or 8,000 of search effort. At 4,000 km of search effort the encounter process alone generated unbiased and precise estimates (CV = 0.18, RB = -0.01) and additional information did not improve model performance (e.g., encounter, harvest and 8 collars: CV = 0.13, RB = -0.04).

### Combining increased effort and additional information

**Fig 7:**
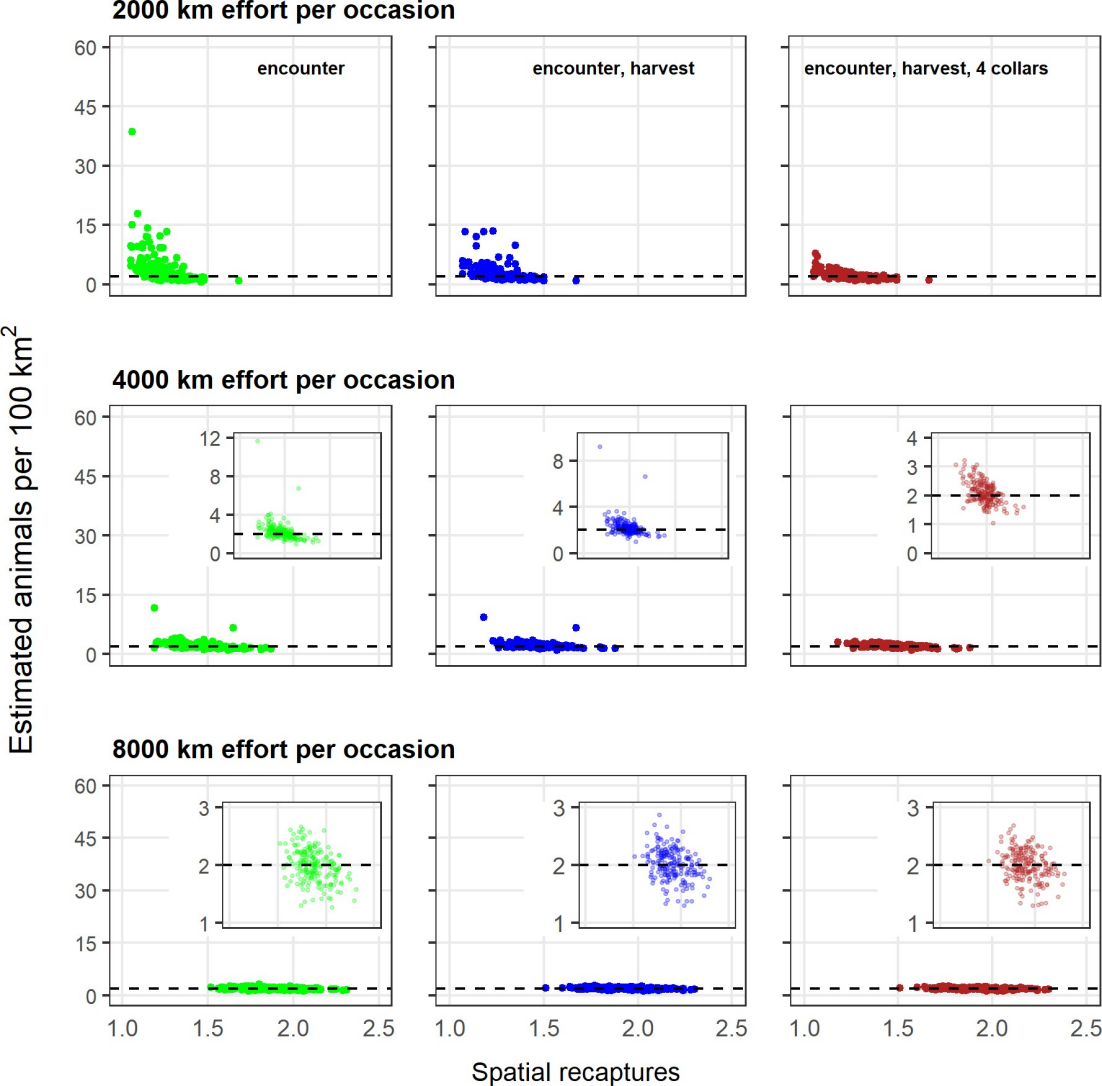
Density estimates as a function of spatial recaptures. The relationship between the underlying median number of spatial recaptures reflecting three levels of effort (uncorrelated), and the resulting density estimates for three models that incorporated increasing amounts of information. The inset graphs show the same data with a y-axis that is rescaled to better represent estimates. Results from a model that included telemetry information from 8 collared individuals were omitted for clarity. The true density was 2 individuals per 100 km^2^.

Model performance improved in the presence of increased effort and/or additional sources of information (Fig 4, S1 Table). Low effort generated sparse data sets with comparatively few spatial recaptures of individuals and resulted in biased and imprecise estimates of density (Fig 7). Additional information improved estimates for these scenarios with low effort, though density estimates remained biased. However, as effort and spatial recaptures increased, the addition of harvest or telemetry information provided less benefit in terms of reducing bias or increasing precision as the observation process alone generated rates of spatial recaptures sufficient to estimate density (Figs 4 and 7). This overall pattern was the same at higher animal densities (*N* = 400, 4 individuals per 100 km^2^) (S2 Table, S3 Fig).

## Discussion

Our results demonstrate that these models perform well in the presence of multiple sources of heterogeneity given sufficient information on animal movement. Model performance consistently improved as higher effort resulted in denser data sets and/or as additional information was included from harvested individuals or telemetered individuals. However, the improvement in model performance consistently diminished with increasing effort such that reliable estimates were generated both from medium levels of effort with information from harvest and telemetry as well as from high levels of effort and no additional information from harvest or telemetry. More informative data sets also moderated the consistent positive bias introduced when search effort was correlated with the density, a source of bias particular to unstructured spatial sampling. This approach to assessing model performance given variable effort and sources of additional information could be used to develop logistically feasible and cost-effective sampling scenarios that yield accurate and precise estimates of density.

SCR models have improved the ability to estimate abundance and density due, in part, to the explicit incorporation of the spatial arrangement of individuals and their variable exposure to encounter, as well as the flexible model formulation that facilitates the integration of different data sources [28,32,34]. Yet, similar to non-spatial methods, estimation in a SCR framework requires sufficient information to yield reliable estimates [16,20]. Low effort manifested as sparse data with few captured individuals and few spatial recaptures. Such sparse data sets containing little information on the number of individuals present and the encounter probabilities of those individuals in the trapping grid were unable to accurately estimate the parameters of the SCR model and resulted in biased and imprecise density estimates. This problem associated with sparse data was ameliorated as the information in data sets improved with higher effort and/or additional sources of information. Thus, we can confirm previous suggestions of the reliability of SCR models, even in the presence of multiple sources of heterogeneity, given sufficient information to estimate density [20].

Our work highlights the persistent bias and imprecision of estimates based on sparse data, suggesting it is important to consider how effort translates into encounters for field studies using spatially unstructured sampling. For SCR models, bias can result from both unmodeled heterogeneity and low probabilities of encounter that yield sparse data. Unmodeled heterogeneity in encounter, e.g., among-individual differences in encounter arising from age, social status, sex, or individual differences in space use, typically results in negatively biased and overly precise estimates of density [17–19]. In contrast to the bias introduced by model mis-specification, low probabilities of encounter and sparse data have been shown to consistently result in overestimates of density in both empirical [36] and simulation-based studies [16]. Our simulations did not address the consequences of model mis-specification and are thus consistent with the latter by demonstrating that sufficiently high probabilities of encounter (translating into larger sample sizes and more spatial recaptures) yield unbiased and precise estimates for a correctly specified model, e.g. 4,000 km of effort resulted in reasonable estimates and 8,000 km of effort resulted in unbiased and very precise estimates. Yet, a correlation between search effort and animal density is a particular problem for spatially unstructured sampling designs [25,41] and is a persistent source of positive bias in density estimates. However, our results suggest that the magnitude of this bias relative to estimates from uncorrelated search effort diminishes with higher effort and is less significant at higher animal densities. The relationship between effort, encounter, data sparsity and the resulting density estimates is a complex interaction between ecological (e.g., population density and spatial arrangement on the landscape) and encounter processes (e.g., detector efficiency, field conditions, and logistical limitations) such that we cannot make general recommendations for levels of effort required to generate unbiased estimates for other studies using spatially unstructured sampling. Moreover, the precision required for management decisions is likely area- and species-specific. However, we can recommend a similar simulation-based approach that incorporates empirical results be used to understand the system-specific relationship between effort and the reliability of density estimates to inform study design and yield reliable density results. We echo the advice that pilot studies will likely be needed in most cases to inform study design [45].

The ability to incorporate additional sources of information to aid model estimation is a strength of the flexible formulation of SCR models, but the magnitude of the benefit for density estimation is unclear. Our work demonstrates that with sparse data sets, the addition of information from harvested individuals and telemetry can substantially reduce the bias and increase the precision of density estimates to some degree. Reliable density estimates from SCR models depend on accurate estimation of parameters at multiple levels of the model hierarchy, including those governing the spatial point process (those covariates governing the distribution of animals on the landscape), the baseline probability of encounter in each trapping grid cell (those covariates governing the encounter probability if the trap center overlies the activity center of an animal), and the attenuation of encounter probability with distance from an activity center [8,13,14]. Whereas the addition of a few harvested individuals can help inform all parameters across the model, information from telemetry can only inform the scale parameters that relate distance from activity center to encounter probability. We interpret our results to suggest that the addition of telemetry information greatly aided the estimation of these scale parameters at low effort, but that persistently sparse data failed to provide sufficient information for other parts of the model governing baseline probabilities of encounter, the accurate estimation of which are required for reliable results. Medium effort resulted in more-informative data sets that were better able estimate model parameters, though the addition of telemetry information again improved estimates such that the combination of the two resulted in unbiased and precise estimates. Finally, at high effort there was sufficient information in the encounter data set to estimate all parts of the model such that the addition of telemetry did not appreciably improve results. The important role of extra information in these results is in apparent contrast to previous work that suggested sources of additional information resulted in marginal improvement in density estimates [34]. Rather than a contradiction, we interpret these seemingly disparate results to be consistent. This previous work used a higher baseline probability of encounter, i.e., there was already sufficient information to estimate model parameters and the more accurate information on space use provided by telemetry provided only marginal benefit, very similar to our high effort scenarios. Taken together, our results suggest that field studies can benefit from a practical combination of effort and additional information. In cases where effort has the potential to be limited (e.g., finite resources and logistical constraints, or bad weather preventing search), additional information from another encounter process and a small number of collars can greatly improve the reliability of density estimates. Where additional sources of information are impractical or impossible, increased search effort alone can yield data informative enough to result in similarly reliable estimates.

Our data-generating scenarios and parameter choices were guided by the empirical results of a previous study that used spatially unstructured sampling and SCR models to estimate the density of mountain lions in western Montana. Because of the specificity of our work, our methods cannot be directly applied to other studies aimed at estimating the densities of different species in different areas. Moreover, we used a modest number of scenarios to address our goals due, in part, to the computational demands of simulating data from these models. Additional work is needed to assess the consequences of variation in other components of hierarchical SCR models as it relates to study design. However, the work presented here can serve as an example that can be modified as necessary to evaluate and choose study designs for other species- and area-specific circumstances based on what can be learned about interactions between animal density, effort, encounter, data sparsity and the reliability of these models for additional species and areas. Future work on the consequences of model mis-specification, variation in the underlying relationship between animal density and landscape processes, and additional sources of among-individual heterogeneity in encounter and space use would provide more insight on how variation in the observational and ecological processes that generate SCR data impact the robustness of density estimates.

### Conclusions

As the use of SCR models to estimate abundance and density increases, and as the palette of different sampling methodologies and methods to incorporate multiple sources of information diversifies, it is critical to examine the reliability of model estimates in the presence of multiple sources of potential bias and imprecision in density estimates. Given the rarity of SCR model validation against populations of known size, simulation-based approaches are the most practical method to evaluate model performance. Our paradigm of case-study based model validation presented here is quite general. For assessing patterns of variation in animal density across the landscape, SCR models are a reliable and robust tool that may be applied to animal populations that are persistently challenging to sample. However, similar to every other method of estimating abundance, SCR models are not immune to the problems induced by sparse data. The flexible formulation of SCR models allows a practical balance between increased effort and sources of additional information to generate reliable estimates, and our approach should yield practical recommendations for study design.

## Supporting information

S1 Table Summary of density estimates (per 100 km^2^) for each combination of scenario and model. Estimates are summarized using the mean, standard error, 2.5% and 97.5% percentiles, root mean square error (CV) and relative bias (RB). The true density was 2 individuals per 100 km^2^.
(DOCX)

S2 Table Summary of density estimates (per 100 km^2^) for each combination of scenario and model. Estimates are summarized using the mean, standard error, 2.5% and 97.5% percentiles, root mean square error (CV) and relative bias (RB). The true density was 4 individuals per 100 km^2^.
(DOCX)

S1 File R code required to simulate SCR data and estimate model parameters from simulated data sets. (R)

S1 Fig Density estimates and search effort. Estimates of animal density resulting from the encounter process alone for each level of search effort (thin line = 2.5% to 97.5% percentiles; gray rectangle = 25% to 75% percentiles; horizontal line = mean). The true density was 4 individuals per 100 km^2^. The inset graphs show the same data with a y-axis that is rescaled to better represent estimates.
(TIFF)

S2 Fig Density estimates and additional information. Consequences of including additional information on estimates of animal density for each level of search effort that was uncorrelated with animal density (thin line = 2.5% to 97.5% percentiles; gray rectangle = 25% to 75% percentiles; horizontal line = mean). The true density was 4 individuals per 100 km^2^. The inset graphs show the same data with a y-axis that is rescaled to better represent estimates.
(TIFF)

S3 Fig Density estimates as a function of spatial recaptures. The relationship between the underlying median number of spatial recaptures reflecting three levels of effort (uncorrelated), and the resulting density estimates for three models that incorporated increasing amounts of information. The inset graphs show the same data with a y-axis that is rescaled to better represent estimates. Results from a model that included telemetry information from 8 collared individuals were omitted for clarity. The true density was 4 individuals per 100 km^2^.
(TIFF)

## References

[1] Seber GAF. The estimation of animal abundance and related parameters. 1982;

[2] Nichols JD. Capture-recapture models. BioScience. 1992;42(2):94–102.

[3] Williams BK, Nichols JD, Conroy MJ. Analysis and management of animal populations. Academic Press; 2002.

[4] Royle JA, Dorazio RM. Hierarchical modeling and inference in ecology: the analysis of data from populations, metapopulations and communities. Elsevier; 2008.

[5] MacKenzie DI, Nichols JD, Sutton N, Kawanishi K, Bailey LL. Improving inferences in population studies of rare species that are detected imperfectly. Ecology. 2005;86(5):1101–1113.

[6] Kery M, Gardner B, Stoeckle T, Weber D, Royle JA. Use of spatial capture-recapture modeling and DNA data to estimate densities of elusive animals. Conserv Biol. 2011;25(2):356–364. 10.1111/j.1523-1739.2010.01616.x 21166714

[7] Boitani L, Powell RA. Carnivore ecology and conservation: a handbook of techniques. Oxford University Press; 2012.

[8] Efford M. Density estimation in live-trapping studies. Oikos. 2004;106(3):598–610.

[9] Boulanger J, Stenhouse G, Munro R. Sources of heterogeneity bias when DNA mark-recapture sampling methods are applied to grizzly bear (*Ursus arctos*) populations. J Mammal. 2004;85(4):618–624.

[10] Pledger S. The performance of mixture models in heterogeneous closed population capture–recapture. Biometrics. 2005;61(3):868–873. 10.1111/j.1541-020X.2005.00411_1.x 16135042

[11] Ivan JS, White GC, Shenk TM. Using simulation to compare methods for estimating density from capture–recapture data. Ecology. 2013;94(4):817–826.

[12] Rich LN, Kelly MJ, Sollmann R, Noss AJ, Maffei L, Arispe RL, et al Comparing capture-recapture, mark-resight, and spatial mark-resight models for estimating puma densities via camera traps. J Mammal. 2014;95(2):382–391.

[13] Royle JA, Young KV. A hierarchical model for spatial capture–recapture data. Ecology. 2008;89(8):2281–2289. 18724738 10.1890/07-0601.1

[14] Efford MG, Fewster RM. Estimating population size by spatially explicit capture–recapture. Oikos. 2013;122(6):918–928.

[15] Sollmann R, Gardner B, Belant JL. How does spatial study design influence density estimates from spatial capture-recapture models? PloS One. 2012;7(4):e34575 10.1371/journal.pone.0034575 22539949 PMC3335117

[16] Sun CC, Fuller AK, Royle JA. Trap configuration and spacing influences parameter estimates in spatial capture-recapture models. PloS One. 2014;9(2):e88025 10.1371/journal.pone.0088025 24505361 PMC3914876

[17] Dorazio RM, Royle AJ. Mixture models for estimating the size of a closed population when capture rates vary among individuals. Biometrics. 2003;59(2):351–364. 12926720 10.1111/1541-0420.00042

[18] Howe EJ, Obbard ME, Kyle CJ. Combining data from 43 standardized surveys to estimate densities of female American black bears by spatially explicit capture–recapture. Popul Ecol. 2013;55(4):595–607.

[19] Gerber BD, Parmenter RR. Spatial capture–recapture model performance with known small-mammal densities. Ecol Appl. 2015;25(3):695–705. 26214915 10.1890/14-0960.1

[20] Keiter DA, Davis AJ, Rhodes OE, Cunningham FL, Kilgo JC, Pepin KM, et al Effects of scale of movement, detection probability, and true population density on common methods of estimating population density. Sci Rep. 2017;7(1):9446 10.1038/s41598-017-09746-5 28842589 PMC5573344

[21] Sharma RK, Jhala Y, Qureshi Q, Vattakaven J, Gopal R, Nayak K. Evaluating capture–recapture population and density estimation of tigers in a population with known parameters. Anim Conserv. 2010;13(1):94–103.

[22] Després-Einspenner M-L, Howe EJ, Drapeau P, Kühl HS. An empirical evaluation of camera trapping and spatially explicit capture-recapture models for estimating chimpanzee density. Am J Primatol. 2017;79(7):e22647. 10.1002/ajp.22647 28267880

[23] Gardner B, Royle JA, Wegan MT, Rainbolt RE, Curtis PD. Estimating black bear density using DNA data from hair snares. J Wildl Manag. 2010;74(2):318–325.

[24] Sun CC, Fuller AK, Hare MP, Hurst JE. Evaluating population expansion of black bears using spatial capture-recapture. J Wildl Manag. 2017 7o 1;81(5):814–23.

[25] Thompson CM, Royle JA, Garner JD. A framework for inference about carnivore density from unstructured spatial sampling of scat using detector dogs. J Wildl Manag. 2012;76(4):863–871.

[26] Fuller AK, Sutherland CS, Royle JA, Hare MP. Estimating population density and connectivity of American mink using spatial capture–recapture. Ecol Appl. 2016;26(4):1125–1135. 27509753 10.1890/15-0315

[27] López-Bao JV, Godinho R, Pacheco C, Lema FJ, García E, Llaneza L, et al Toward reliable population estimates of wolves by combining spatial capture-recapture models and non-invasive DNA monitoring. Sci Rep. 2018;8(1):2177 10.1038/s41598-018-20675-9 29391588 PMC5794931

[28] Sollmann R, Gardner B, Chandler RB, Shindle DB, Onorato DP, Royle JA, et al Using multiple data sources provides density estimates for endangered Florida panther. J Appl Ecol. 2013;50(4):961–968.

[29] Royle JA, Chandler RB, Sollmann R, Gardner B. Spatial capture-recapture. Academic Press; 2013.

[30] Chandler RB, Clark JD. Spatially explicit integrated population models. Methods Ecol Evol. 2014;5(12):1351–1360.

[31] Borchers DL, Stevenson BC, Kidney D, Thomas L, Marques TA. A unifying model for capture–recapture and distance sampling surveys of wildlife populations. J Am Stat Assoc. 2015;110(509):195–204. 10.1080/01621459.2014.893884 26063947 PMC4440664

[32] Linden DW, Sirén AP, Pekins PJ. Integrating telemetry data into spatial capture–recapture modifies inferences on multi-scale resource selection. Ecosphere. 2018;9(4). 10.1002/ecs2.2188

[33] Proffitt KM, Goldberg JF, Hebblewhite M, Russell R, Jimenez BS, Robinson HS, et al Integrating resource selection into spatial capture-recapture models for large carnivores. Ecosphere. 2015;6(11):1–15.

[34] Royle JA, Chandler RB, Sun CC, Fuller AK. Integrating resource selection information with spatial capture–recapture. Methods Ecol Evol. 2013;4(6):520–530.

[35] Tenan S, Pedrini P, Bragalanti N, Groff C, Sutherland C. Data integration for inference about spatial processes: A model-based approach to test and account for data inconsistency. PloS One. 2017;12(10):e0185588 10.1371/journal.pone.0185588 28973034 PMC5626469

[36] Jŭnek T, Vymyslická PJ, Hozdecká K, Hejcmanová P. Application of spatial and closed capture-recapture models on known population of the Western Derby eland (*Taurotragus derbianus derbianus*) in Senegal. PloS One. 2015;10(9):e0136525 10.1371/journal.pone.0136525 26334997 PMC4559471

[37] Robinson HS, Ruth T, Gude JA, Choate D, DeSimone R, Hebblewhite M, et al Linking resource selection and mortality modeling for population estimation of mountain lions in Montana. Ecol Model. 2015;312:11–25.

[38] Robert CP, Casella G, Casella G. Introducing monte carlo methods with r. Vol. 18 New York: Springer; 2010.

[39] Logan KA, Irwin LL, Skinner R. Characteristics of a hunted mountain lion population in Wyoming. J Wildl Manag. 1986;50(4):648–654.

[40] Elbroch LM, Lendrum PE, Quigley H, Caragiulo A. Spatial overlap in a solitary carnivore: support for the land tenure, kinship or resources dispersion hypotheses? J Anim Ecol. 2016;85: 487–496. 10.1111/1365-2656.12447 26395576

[41] Russell RE, Royle JA, Desimone R, Schwartz MK, Edwards VL, Pilgrim KP, et al Estimating abundance of mountain lions from unstructured spatial sampling. J Wildl Manag. 2012;76(8):1551–1561.

[42] Sutherland C, Royle JA, Linden D. oSCR: multi-session sex-structured spatial capture-recapture models. R Package Version 030 0. 2016;

[43] R Core Team. R: A Language and Environment for Statistical Computing [Internet]. Vienna, Austria: R Foundation for Statistical Computing; 2018 Available from: https://www.R-project.org/

[44] de Valpine P, Turek D, Paciorek CJ, Anderson-Bergman C, Lang DT, Bodik R. Programming with models: writing statistical algorithms for general model structures with NIMBLE. J Comput Graph Stat. 2017;26(2):403–413.

[45] Mumma MA, Zieminski C, Fuller TK, Mahoney SP, Waits LP. Evaluating noninvasive genetic sampling techniques to estimate large carnivore abundance. Mol Ecol Resour. 2015;15(5):1133–1144. 10.1111/1755-0998.12390 25693632

