# Supplementary material for: Simulation-based validation of spatial capture-recapture models: A case study using mountain lions
Source: PLoS One. 2019 Apr 19;14(4):e0215458. doi: 10.1371/journal.pone.0215458 (PMC6474654; doi:10.1371/journal.pone.0215458)
Supplement: S1 Table — Estimates are summarized using the mean, standard error, 2.5% and 97.5% percentiles, root mean square error (CV) and relative bias (RB). The true density was 2 individuals per 100 km2. (DOCX) [file pone.0215458.s001.docx]

|  | **2000 km effort per occasion** | | **4000 km effort per occasion** | | **8000 km effort per occasion** | |
| --- | --- | --- | --- | --- | --- | --- |
|  | uncorrelated | correlated | uncorrelated | correlated | uncorrelated | correlated |
|  | Scenario 1 | Scenario 2 | Scenario 3 | Scenario 4 | Scenario 5 | Scenario 6 |
| **Encounter alone** | | | | | | |
| mean | 3.68 | 3.24 | 2.21 | 2.36 | 2.00 | 2.05 |
| se | 4.08 | 3.12 | 0.93 | 1.13 | 0.29 | 0.27 |
| 2.5%, 97.5% | 0.97, 13.06 | 1.28, 14.12 | 1.33, 3.77 | 1.54, 3.62 | 1.42, 2.58 | 1.56, 2.59 |
| CV | 1.11 | 0.96 | 0.42 | 0.48 | 0.15 | 0.13 |
| RB | 0.84 | 0.62 | 0.11 | 0.18 | 0.00 | 0.03 |
| **Encounter, harvest** | | | | | | |
| mean | 2.78 | 2.96 | 2.23 | 2.24 | 2.02 | 2.05 |
| se | 2.06 | 2.60 | 0.73 | 0.53 | 0.27 | 0.26 |
| 2.5%, 97.5% | 1.13, 9.83 | 1.34, 8.38 | 1.50, 3.35 | 1.60, 3.36 | 1.40, 2.55 | 1.56, 2.58 |
| CV | 0.74 | 0.88 | 0.33 | 0.24 | 0.13 | 0.13 |
| RB | 0.39 | 0.48 | 0.11 | 0.12 | 0.01 | 0.03 |
| **Encounter, harvest, 4 collars** | | | | | | |
| mean | 2.16 | 2.28 | 2.10 | 2.13 | 2.00 | 2.02 |
| se | 0.90 | 0.71 | 0.38 | 0.35 | 0.26 | 0.25 |
| 2.5%, 97.5% | 1.03, 4.41 | 1.13, 3.78 | 1.45, 2.97 | 1.54, 2.87 | 1.45, 2.49 | 1.54, 2.53 |
| CV | 0.42 | 0.31 | 0.18 | 0.17 | 0.13 | 0.13 |
| RB | 0.08 | 0.14 | 0.05 | 0.06 | 0.00 | 0.01 |
| **Encounter, harvest, 8 collars** | | | | | | |
| mean | 2.14 | 2.27 | 2.08 | 2.11 | 1.99 | 2.02 |
| se | 0.87 | 0.72 | 0.38 | 0.34 | 0.26 | 0.26 |
| 2.5%, 97.5% | 1.05, 4.56 | 1.17, 3.90 | 1.42, 2.87 | 1.56, 2.84 | 1.45, 2.47 | 1.53, 2.55 |
| CV | 0.40 | 0.32 | 0.18 | 0.16 | 0.13 | 0.13 |
| RB | 0.07 | 0.13 | 0.04 | 0.06 | 0.00 | 0.01 |
