# Supplementary material for: Simulation-based validation of spatial capture-recapture models: A case study using mountain lions
Source: PLoS One. 2019 Apr 19;14(4):e0215458. doi: 10.1371/journal.pone.0215458 (PMC6474654; doi:10.1371/journal.pone.0215458)
Supplement: S2 Table — Estimates are summarized using the mean, standard error, 2.5% and 97.5% percentiles, root mean square error (CV) and relative bias (RB). The true density was 4 individuals per 100 km2. (DOCX) [file pone.0215458.s002.docx]

|  | **2000 km effort per occasion** | | **4000 km effort per occasion** | | **8000 km effort per occasion** | |
| --- | --- | --- | --- | --- | --- | --- |
|  | uncorrelated | correlated | uncorrelated | correlated | uncorrelated | correlated |
|  | Scenario 1 | Scenario 2 | Scenario 3 | Scenario 4 | Scenario 5 | Scenario 6 |
| **Encounter alone** | | | | | | |
| mean | 4.95 | 5.25 | 3.95 | 4.3 | 3.91 | 4.11 |
| se | 3.43 | 3.93 | 0.71 | 0.75 | 0.40 | 0.35 |
| 2.5%, 97.5% | 2.24, 14.5 | 2.82, 12.6 | 2.81, 5.54 | 3.20, 6.05 | 3.22, 4.72 | 3.42, 4.68 |
| CV | 0.69 | 0.75 | 0.18 | 0.18 | 0.10 | 0.09 |
| RB | 0.24 | 0.31 | -0.01 | 0.07 | -0.02 | 0.03 |
| **Encounter, harvest** | | | | | | |
| mean | 4.85 | 4.72 | 4.00 | 4.26 | 3.95 | 4.12 |
| se | 3.43 | 1.73 | 0.62 | 0.67 | 0.37 | 0.34 |
| 2.5%, 97.5% | 2.24, 12.30 | 2.98, 8.50 | 3.00, 5.54 | 3.28, 5.79 | 3.26, 4.64 | 3.41, 4.75 |
| CV | 0.54 | 0.38 | 0.16 | 0.16 | 0.09 | 0.08 |
| RB | 0.21 | 0.18 | 0.0 | 0.07 | -0.01 | 0.03 |
| **Encounter, harvest, 4 collars** | | | | | | |
| mean | 4.11 | 4.28 | 3.89 | 4.15 | 3.92 | 4.08 |
| se | 1.15 | 0.89 | 0.52 | 0.57 | 0.36 | 0.33 |
| 2.5%, 97.5% | 2.44, 6.44 | 2.91, 6.31 | 2.96, 5.03 | 3.20, 5.35 | 3.26, 4.56 | 3.43, 4.64 |
| CV | 0.28 | 0.21 | 0.13 | 0.14 | 0.09 | 0.08 |
| RB | 0.03 | 0.07 | -0.03 | 0.04 | -0.02 | 0.02 |
| **Encounter, harvest, 8 collars** | | | | | | |
| mean | 4.07 | 4.28 | 3.86 | 4.15 | 3.92 | 4.08 |
| se | 1.09 | 0.90 | 0.51 | 0.58 | 0.36 | 0.34 |
| 2.5%, 97.5% | 2.45, 6.51 | 2.89, 6.24 | 2.93, 5.06 | 3.23, 5.37 | 3.24, 4.58 | 3.45, 4.69 |
| CV | 0.27 | 0.21 | 0.13 | 0.14 | 0.09 | 0.08 |
| RB | 0.02 | 0.07 | -0.04 | 0.04 | -0.02 | 0.02 |
