# Supplementary figures and images for: Simulation-based validation of spatial capture-recapture models: A case study using mountain lions
Source: PLoS One. 2019 Apr 19;14(4):e0215458. doi: 10.1371/journal.pone.0215458 (PMC6474654; doi:10.1371/journal.pone.0215458)

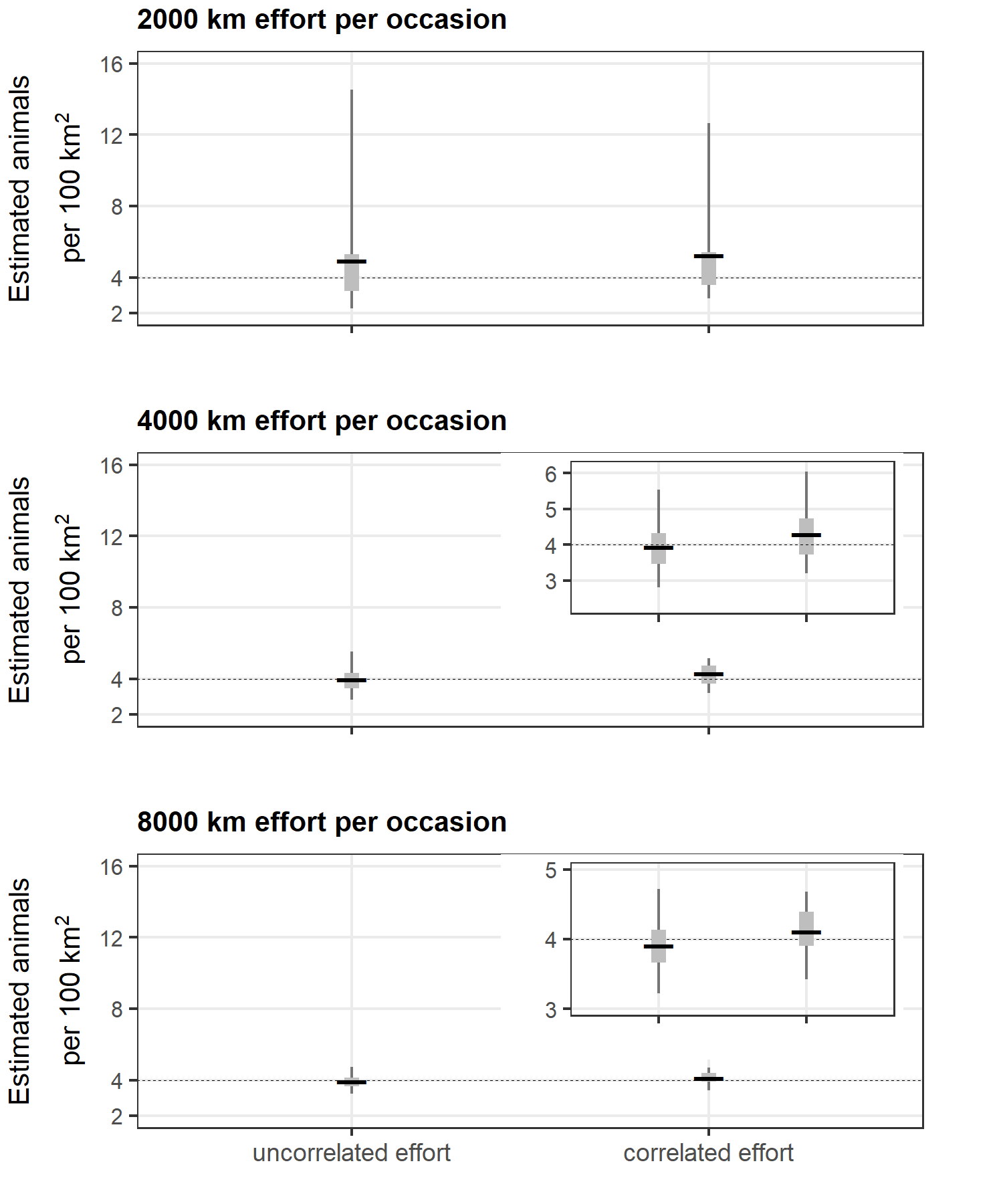

Supplement: S1 Fig — Estimates of animal density resulting from the encounter process alone for each level of search effort (thin line = 2.5% to 97.5% percentiles; gray rectangle = 25% to 75% percentiles; horizontal line = mean). The true density was 4 individuals per 100 km2. The inset graphs show the same data with a y-axis that is rescaled to better represent estimates. (TIFF) [file pone.0215458.s004.tiff]

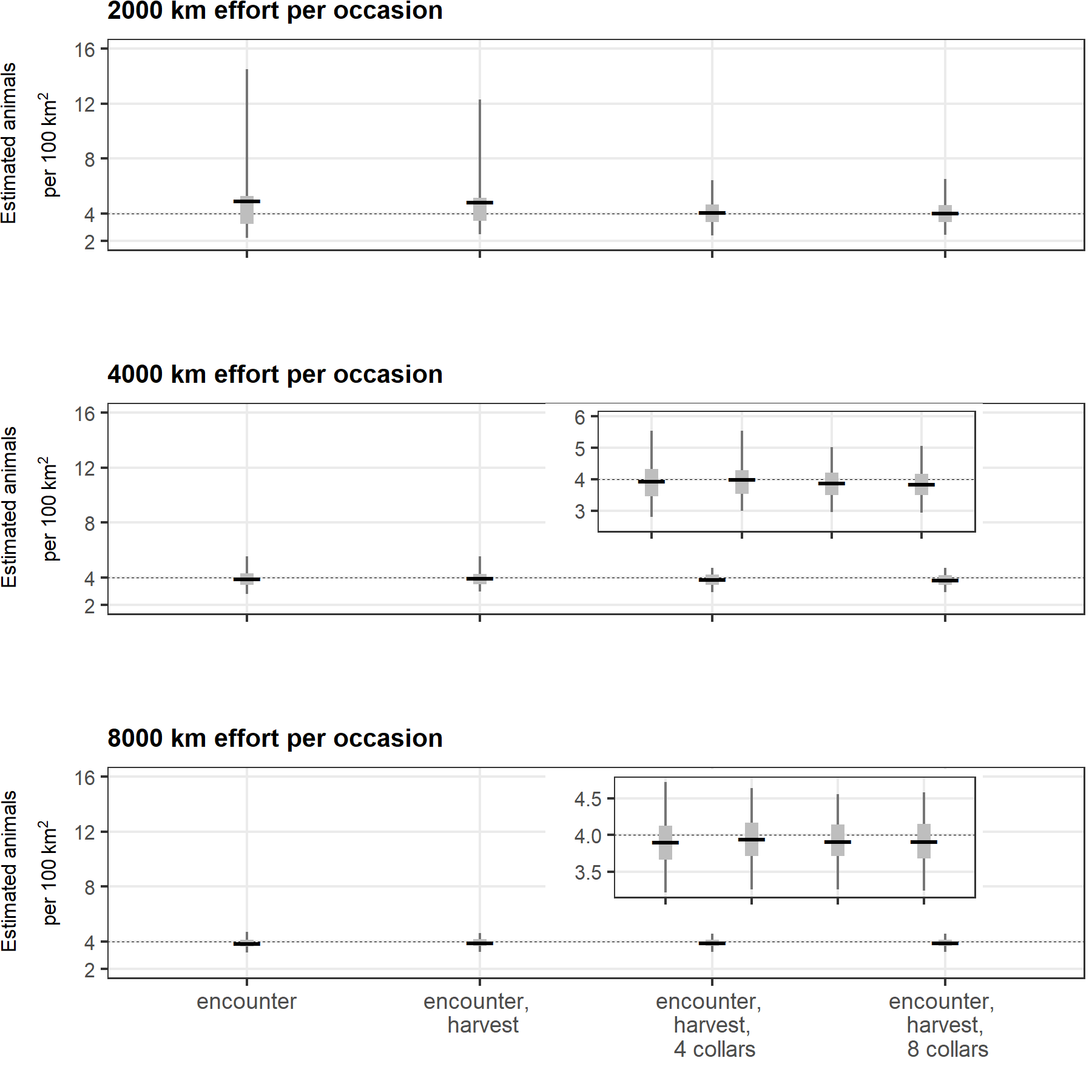

Supplement: S2 Fig — Consequences of including additional information on estimates of animal density for each level of search effort that was uncorrelated with animal density (thin line = 2.5% to 97.5% percentiles; gray rectangle = 25% to 75% percentiles; horizontal line = mean). The true density was 4 individuals per 100 km2. The inset graphs show the same data with a y-axis that is rescaled to better represent estimates. (TIFF) [file pone.0215458.s005.tiff]

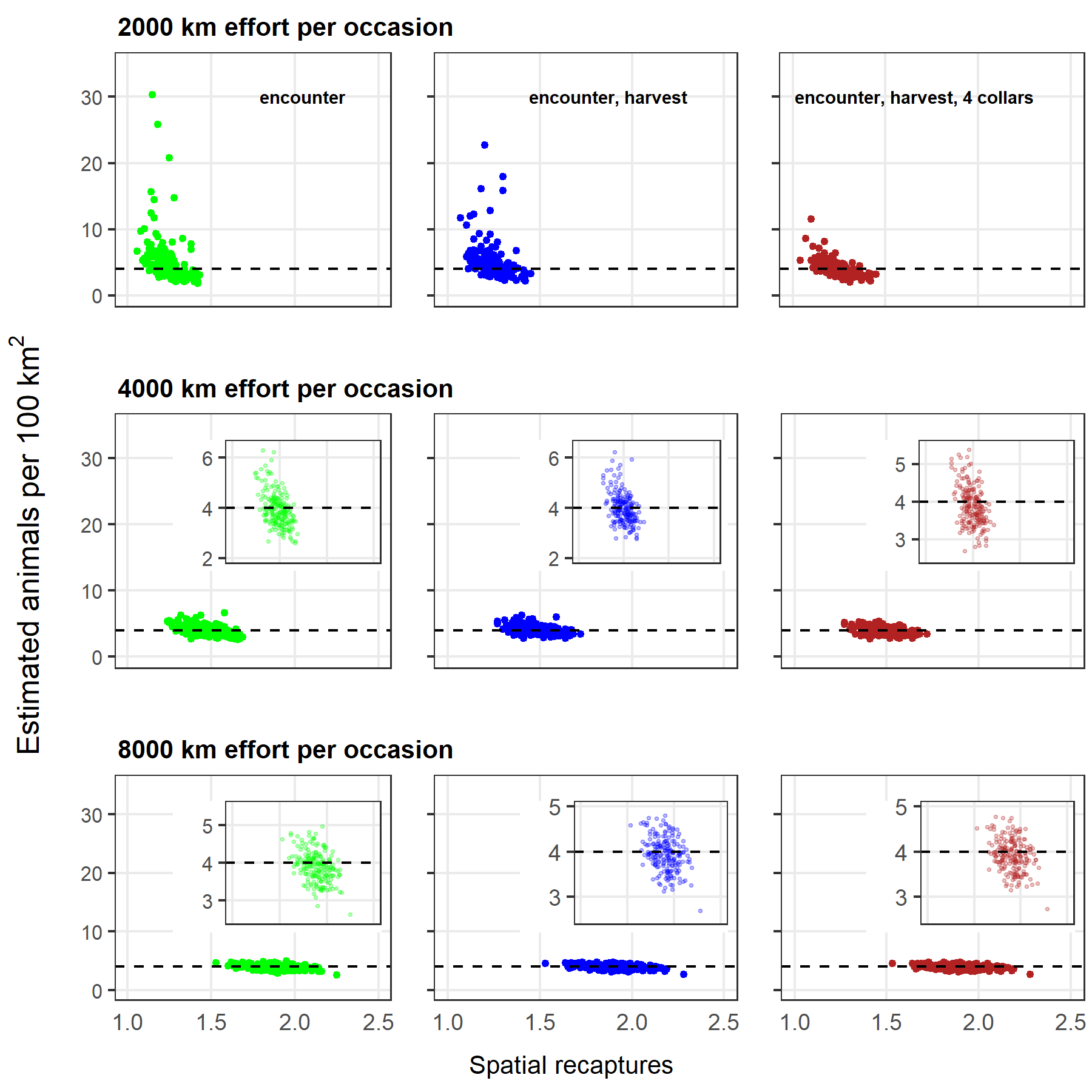

Supplement: S3 Fig — The relationship between the underlying median number of spatial recaptures reflecting three levels of effort (uncorrelated), and the resulting density estimates for three models that incorporated increasing amounts of information. The inset graphs show the same data with a y-axis that is rescaled to better represent estimates. Results from a model that included telemetry information from 8 collared individuals were omitted for clarity. The true density was 4 individuals per 100 km2. (TIFF) [file pone.0215458.s006.tiff]
